# Role of Posterior Carbon Fiber Implants in Spine Tumor Surgery

**DOI:** 10.1177/21925682241259778

**Published:** 2025-01-12

**Authors:** Christopher A. Alvarez-Breckenridge, Robert North, Claudio Tatsui, Naresh Kumar, Sheng-fu Lo, Karim Mohammed, Jeremy Reynolds, Aron Lazary, Ilya Laufer, Jorrit Jan Verlaan, Ziya Gokaslan, Alessandro Luzzati, Riccardo Cecchinato, John Shin, Francis Hornicek, Alexander Disch, Matthew Goodwin, Rory Goodwin, Arjun Sahgal, Alessandro Gasbarrini, Stefano Boriani

**Affiliations:** 1 Department of Neurosurgery, 4002 The University of Texas MD Anderson Cancer Center, Houston, TX, USA; 2 Department of Orthopaedic Surgery, 150744 National University Health System, Singapore, Singapore; 3 Department of Neurosurgery, 232890 Donald and Barbara Zucker School of Medicine at Hofstra/Northwell, Great Neck, NY, USA; 4 Department of Orthopedics, 6915 Mayo Clinic, Rochester, MN, USA; 5 Department of Spinal Division, 6397 Oxford University Hospitals NHS Trust, Oxford, UK; 6 Department of Spine Surgery, Semmelweis University, Budapest, Hungary; 7 Department of Neurosurgery, 12297 NYU Langone Medical Center, New York, NY, USA; 8 Department of Orthopaedics, 8124 UMC Utrecht, Utrecht, Netherlands; 9 Department of Spine, 6752 Brown University, Providence, RI, USA; 10 Department of Orthopedics, 46767 IRCCS Galeazzi Orthopaedic Institute, Milan, Italy; 11 Department of Neurosurgery, 2348 Massachusetts General Hospital, Boston, MA, USA; 12 Department of Orthopaedics, 12235 University of Miami Miller School of Medicine, Miami, FL, USA; 13 Department of Orthopedics, Medical University at Dresden, Dresden, Germany; 14 Department of Orthopaedic Surgery, 7548 Washington University, St. Louis, MO, USA; 15 Department of Neurosurgery, 22957 Duke University Hospital, Durham, NC, USA; 16 Department of Radiation Oncology, 71545 Sunnybrook Health Sciences Centre, Toronto, ON, CA; 17 Department of Spine Surgery, 18509 Institutio Ortopedico Rizzoli di Bolgna, Bologna, Italy

**Keywords:** pedicle screw, tumor, carbon fiber

## Abstract

**Study Design:**

Narrative Review.

**Objective:**

The management of spinal tumors requires a multi-disciplinary approach including surgery, radiation, and systemic therapy. Surgical approaches typically require posterior segmental instrumentation to maintain long-term spinal stability. Carbon fiber reinforced pedicle screws (CFRP) are increasingly used in the oncologic setting due to reductions in both imaging artifacts and radiotherapy perturbations compared to titanium implants. We performed a review of the literature and highlight advantages and future areas of study for CFRP.

**Methods:**

We performed a systematic review of the literature using the Preferred Reporting Items for Systematic Reviews and Meta-Analyses guidelines and identified 10 articles including 573 patients. Across all studies we reviewed patient demographics, tumor types treated, hardware-related features, complication rates, recurrence, survival, and follow-up.

**Results:**

Across 10 studies, a total of 1371 screws placed. Surgical and non-surgical complications were reported in 18.3% of patients. Disease progression at the surgical site was detected in 7.3% of patients. There was no significant difference in clinical or hardware complications between CFRP or titanium implants. The most frequent complication attributable to implanted CFRP hardware included screw breakage in 2.4% and loosening in 1.7% of patients, respectively.

**Conclusion:**

CFRP provide a unique tool in the setting of spinal oncology. With a safety profile comparable to titanium, we review the documented advantages of CFRP posterior implants compared to titanium, while also addressing their current limitations. Additionally, we highlight several areas of future research to identify the optimal patients who will achieve the greatest benefit of CFRP.

## Introduction

The presence of tumors in the spine reflects both metastatic seeding and the development of primary osseous tumors. Spinal metastases are found in 30%–90% of patients who die of cancer. Their incidence has increased due to improved systemic agents, enhanced imaging modalities that detect tumor formation at earlier time points, and an aging population.^1^ Indications for surgical intervention in the metastatic setting include neurologic compromise, intractable pain, and spinal instability. Additionally, surgical resection, typically requiring spinal instrumentation, followed by adjuvant radiation can be useful for metastatic lesions causing spinal cord compression.^2^ In contrast to spinal metastases, primary bone tumors of the spine represent less than 5% of all bone tumors^3^ and are frequently symptomatic on initial presentation. The approach towards surgical resection for these primary tumors varies on the nature of the tumor, its location, and prior therapies. For the treatment naïve primary tumors of the spine, an en-bloc resection is preferred to achieve improved overall survival and the possibility of a cure.^4,5^ For primary spine tumors that are not amenable to such a surgical approach, high-grade malignancies, or for recurrent lesions, the role of surgery is typically palliative and typically serves as a bridge towards adjuvant radiation and systemic therapy.^6,7^ For both metastatic and primary spinal tumors, the goals of surgery may include decompressing the neural elements, achieving cytoreduction, reducing mechanical pain, treating spinal instability, and preventing or correcting spinal deformity. Since the structural integrity of the spine can be compromised by both spinal tumor burden and surgical decompression, it is critical to stabilize the spine while also planning for adjuvant radiation and radiographic monitoring.

Posterior spinal stabilization has traditionally been achieved with titanium pedicle screws. Despite their widespread use, they have possible limitations in the oncologic setting. These include varying degrees of radiographic artifact on both computed tomography (CT) and magnetic resonance imaging (MRI). While there have been developments in metal suppression protocols for CT and MRI, this artifact may impede monitoring for radiographic evidence of tumor recurrence and impair proper delineation of tumor and neural structures during radiation planning.^8^ Additionally, adjuvant radiation is a critical component for the post operative treatment of spinal metastases and many primary tumors of the spine. However, the presence of metallic implants can have a deleterious effect on the planning and safe delivery of post-operative radiation therapy, particularly when heavy particles are used, due to uneven dose distribution and an associated risk to nearby tissue.^5^

To overcome the mentioned limitations of titanium posterior spinal instrumentation, carbon fiber-reinforced pedicle screws (CFRP) have been increasingly utilized in the oncologic setting due to their reduced metallic artifact, minimal radiation absorption, and potential to improve long-term outcomes for patients with spinal malignancies. In order to achieve spinal stabilization, CFRP screws have biomechanical properties that are comparable to titanium constructs.^9,10^ From an oncologic perspective, the radiolucency of CFRP screws has the potential to improve long-term outcomes when compared to titanium screws due to their reduced interference with post-operative surveillance imaging, improved radiation planning and dose delivery. However, no formal studies have been performed to assess these perceived advantages.^11^ Despite their use at various international centers, ongoing research is needed to prospectively compare the perceived benefits of CFRP instrumentation when compared to titanium, carry out long-term follow-up, identify optimal clinical scenarios for utilization, explore the financial implications of this novel technology, and evaluate the differences between different CFRP systems. In this review we will summarize the clinical reports of CFPR posterior spinal implants, discuss their advantages and drawbacks, and identify future areas of investigation.

## Review of the Literature

A systematic literature review was performed using the Preferred Reporting Items for Systematic Reviews and Meta-Analyses guidelines. The PubMed MEDLINE (National Library of Medicine) databases was utilized from database inception to June 2023. The search was designed to retrieve relevant studies detailing the clinical outcomes of CFRP posterior spinal implants in the surgical treatment of spinal tumors. The following terms were included in the search: “Carbon fiber” AND “implants” AND “spinal” AND “oncology”, “Carbon fiber” AND “implants” AND “spine” AND “oncology” and “Carbon fiber” AND “implants” AND “spine” AND “tumor”. After exclusion of duplicates, a total of 38 unique articles were returned in our search. After screening the abstracts, a total of 10 articles were included for further study^6,11-19^ (Table 1). Inclusion criteria included articles written in English, patients with a diagnosis of spinal tumors requiring spine surgery with the use of posterior CFRP instrumentation, and reports including 5 or more patients. Exclusion criteria included case reports, reviews, editorials, and phantom studies.

**Table 1.** PRISMA Flow Diagram Displaying the Number of Articles Excluded and Included in the Systematic Review.

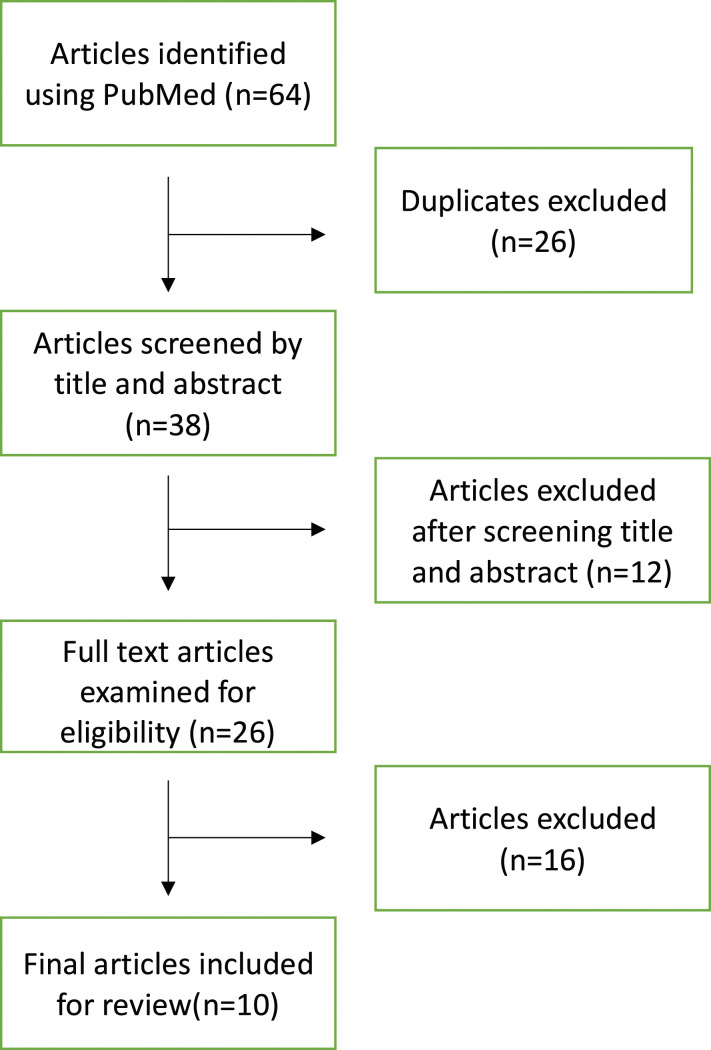

Amongst the 10 studies that were included (Table 2), a total of 573 patients were treated with CFRP instrumentation. The mean age amongst the patients in these studies was 57 years. Both metastatic and primary tumors were represented (80.6% and 19.4% respectively). The number of levels included in the CFRP construct was generally between three and 8. There was a total of 1371 screws placed amongst the reporting studies. For studies reporting the vendor of CFRP implants, VADER (Icotec, Altstatten, Switzerland) was used in 3 studies while Carboclear (CarboFix Orthopedics, Herziliya, Israel) was used in 5 studies. Total surgical and non-surgical complications were reported in 18.3% of patients. Disease progression at the surgical site was detected in 7.3% of patients. However, the literature did not clearly distinguish whether this was secondary to progressive symptoms or enhanced visualization due to radiolucent carbon fiber implants. The median range of follow-up was 10.7 months and overall survival rate was 75.7%.

**Table 2.** Summary of included articles in systematic review of the literature.

Article	Number of Patients	Age	Tumor Types	Number of Levels	Total Screws	Vendor	Complication Rate	Patient with Recurrence	Follow- up	Survival
Mastella et al. (2017)	9		Primary = 9		8 (mean per patient)	Carboclear				
Ringel et al. (2017)	35	63.6	Met = 31, Primary = 4	3 to 7	251	Icotec				
Tedesco et al. (2017)	22	55.9	Primary = 22	3 to 8	167	Carboclear	Screw breakage (n = 1), Loosening (n = 1)	6		
Boriani et al. (2018)	34	57.4	Met = 14,Primary = 20	3 to 8	232	Carboclear	Screw breakage (n = 1), Loosening (n = 2)	6		
Boriani et al. (2020)	6	43.8	Primary = 6					2	31.5	
Cofano et al. (2020)	36	62.2	Met = 36		230	Carboclear	CSF leak (n = 1), Wound dehiscence (n = 1)	2	11	32 of 36
Neal et al. (2021)	28	60	Met = 23,Primary = 5	3 to 8		Carboclear		3	6.5	17 of 28
Shen et al. (2021)	13	57.5	Met = 5, Primary = 8			Carboclear		1	7	11 of 13
Joerger et al. (2022)	321	65	Met = 306, Primary = 15	3 to 7		Icotec	Intraoperative: Durotomy leak (n = 15), Screw breakage (n = 11), Cement extravasation (n = 4); Post- operative: Epidural hematoma (n=6), Surgical site infection (n=15), CSF leak (n=7), wound healing (n = 18), Screw loosening (n = 7), Titanium rod breakage (n = 1), Screw breakage (n = 1)	10	3.2	
Alvarez-Breckenridge et al. (2023)	69	56	Met = 47, Primary = 22	3 to 8	491	Icotec	Screw stripped during insertion (n = 3), Screw breakage (n = 1), Loosening (n = 4), Surgical site infection (n = 3), Hematoma (n = 1), Durotomy (n = 1)	12	5.4	48 of 69

Amongst the included studies, the work by Joerger et al^13^ represents the largest single center experience with CFRP instrumentation. This study included 321 patients who underwent CFRP posterior instrumentation for metastatic and primary tumors. The patient demographics, oncologic pathologies, and surgical approaches were similar to those reported in the other included studies. The reported intraoperative complication rate was 9.3% and revision surgery was performed in 17.1% of patients. However, implant related complications were rare with screw breakage reported in 3.4% and screw loosening in 2.2% of patients, respectively.

Notably, Cofano et al^19^ performed a retrospective comparative study evaluating patients who received either CFRP (11 month mean follow-up) or titanium (14 month mean follow-up) of posterior instrumentation. There was no significant difference between the two groups in terms of postoperative clinical complications or hardware related complications. Screw loosening was found in one patient undergoing titanium instrumentation while there were no hardware complications in the CFRP group. The reported differences between the two groups were the mean duration of surgery and mean blood loss which were both greater in the CFRP group.

Taken together, these retrospective results provide insights into the safety of CFRP posterior instrumentation and suggest that it possesses a safety profile that is similar to titanium instrumentation. Amongst all studies, the most frequently reported complications attributable to implanted CFRP hardware included screw breakage in 2.4% and loosening in 1.7% of patients, respectively. Regarding the occurrence of screw breakage or stripping during insertion, there appeared to be an association with the quality of bone (e.g., osteoblastic lesions) at the site of screw insertion.^6,11^ Larger studies will be needed to further validate this and provide insights into surgical decision making when surgeons encounter this clinical scenario. For instance, CFRP implants have a modulus of elasticity closer to cortical bone^20^ and require full length tapping into osteoblastic vertebral body lesions to facilitate screw placement.^18^ The remaining reported complications were attributable to the oncologic procedure rather than the presence of CFRP instrumentation, including surgical site infection, CSF leakage, epidural hematoma, impaired wound healing, and bowel obstruction.

The use of post-operative radiation in the setting of primary and metastatic spine tumors is often a critical component of the post-surgical workflow. Alvarez-Breckenridge et al^11^ report post operative radiation in 36% of primary and 61% of metastatic tumors with a median time to radiation of 35 and 30 days, respectively. Amongst patients undergoing radiation, 62% of primary and 79% of metastatic cases underwent a CT myelogram. Consistent with this study, a recent review by Khan et al^21^ reported results of 326 patients who received CFRP implants for primary or metastatic spine tumors and documented 72.5% of patients receiving post-operative radiation.

Apart from achieving spinal stabilization, CFRP posterior instrumentation has a well-documented impact on the quality of post-operative imaging. Ringel et al^6^ reported their experience with screws and CFRP rods that were non-artifact generating while the screw head is titanium and tip is tantalum. Their studies demonstrated a reduction in imaging artifacts for both CT and 1.5T-MRI following CFRP screw placement compared to titanium. However, with 3T-MRI, imaging artifact was observed secondary to the titanium rod and titanium screw head, particularly when greater than four screws were used. The authors proceeded to study the imaging artifact around titanium and CFRP screws and found a 52% reduction in the latter group. Ringel et al and Mastella et al^14^ reached the similar conclusion that the radiation absorbing components of carbon fiber screws are significantly less than titanium implants, and this correlated with reduced artifact. These authors attempted to link these findings with improved postoperative radiation planning. They quantitatively compared the assigned CT-Housfield (CT-HU) values between titanium and CFRP during treatment planning and detected a 4-fold higher value with titanium compared to CFRP. As a result, with assigned CT-HU values of CFRP implants that more adequately matched biologic HU values, this led to improved accuracy of computational radiotherapy dose calculations. Finally, the authors reported that reduced beam perturbations with CFRP compared to titanium ultimately lead to less dose degradation.^14^

## Discussion of Advantages and Drawbacks

There are several perceived advantages of CFRP implants compared to titanium: 1) the CFRP modulus of elasticity, which is a measure of resistance to deformity under stress closely, resembles cortical bone, 2) improved visualization on post-operative imaging to detect tumor recurrence and fusion, 3) improved radiation planning with the ability to delineate the tumor/normal interface and clearly visualize the spinal cord, and 4) enhanced radiation delivery with reduced radiation absorption. However, the clinical relevance and oncologic benefit of these findings is an area of ongoing investigation.

In biomechanical testing, CFRP have been shown to be non-inferior to titanium. Lindtner et al^9^ performed a cadaveric study comparing CFRP and titanium pedicle screws. The resistance of CFRP and titanium screw to loosening following cranio-caudal load cycles was equivalent. Incorporation of cement augmentation was also investigated. This increased the number of load cycles prior to loosening for both groups and reduced angular screw motion in CFRP compared to titanium augmented screws. Additionally, carbon fiber rods have equivalent mean bending yield load, bending cycling capacity, and stiffness compared to titanium rods.^22,23^ Carbon fiber rod and screw constructs have also demonstrated similar^9^ or less.^24^ microscopic loosening compared to titanium and may be used in combination with cement augmentation via fenestrated CFRP to enhance anchorage.^11,13,25^ These and other early experiences suggest that CFRP has low rates of hardware failure.^19^

Several studies have documented the radiographic advantage of CFRP compared to titanium on post-operative imaging. This has been demonstrated with phantom studies in which CFRP implants led to a 90% reduction in imaging artifacts compared to titanium.^26,27^ Work by Fleege et al^26^ demonstrated enhanced visual accessibility of the spinal canal, neuroforamina, surrounding bone, and soft tissue structures in the presence of CFRP compared to titanium. This led to the conclusion that the reduced artifact of CFRP screws resulted in enhanced radiographic accessibility. Consistent with the documented local recurrence rate of 7.3% amongst all patients undergoing carbon fiber screw placement in our systematic review, the lack of artifact leads to the perceived advantage of improved interpretation of post-operative imaging and more sensitive surveillance for tumor progression or recurrence.^16^ However, formal evidence comparing detection rates of tumor recurrence between CFRP and titanium cases are lacking.

The use of metallic implants is challenging for radiation therapy planning and administration due to metallic artifacts and metallic absorption. Dose calculation can be impaired if planning images are excessively distorted secondary to metallic artifacts. While there is significant institutional variation on radiation planning in the post-operative setting, the use of CT myelogram following implantation of titanium hardware is used to facilitate radiation contouring around the spinal cord due the metallic artifact associated with MRI. The recent report by Alvarez-Breckenridge et al^11^ reported the use of CT myelograms following CFRP placement at MD Anderson Cancer Center. They note that CT myelogram is typically obtained after separation surgery, regardless of titanium or CFRP instrumentation. However, their study highlights a gradual change in institutional practice where 35.1% of radiated cases were planned with MRI simulation rather than CT myelogram. These results suggest that the reduced imaging artifact on MRI with CFRP implants may limit the need for post-operative CT myelograms for radiation planning. While the potential benefits of this requires further study, CT myelogram is associated with risks and potential delays in radiation treatment which could be avoided with the use of MRI simulation for radiation planning.

Radiation planning software uses CT-HU values to calculate radiation plans; however, metallic artifact creates HU that are inaccurate. This leads to large regions of images that can be assigned incorrect CT-HU values which impairs predicted radiation dose contouring and calculation. Strongly absorbing materials such as titanium can cause dose enhancement (ie, backscatter) at the interface of bone and metal resulting in loss of radiation distal to the implant and subsequent dose attenuation. This ultimately results in a deviation between the planned and actual delivered dose distribution.^8^ Consistent with this, Nivelsky et al demonstrated a 10% overdose caused by backscatter when photon therapy is used with titanium implants (compared to 0% with CFRP).^28^ Additionally, titanium was associated with a dose attenuation of 30% compared to 5% with CFRP screws.^28^ These results suggest that artifact-reducing, non-absorbing implants such as CFRP can improve the planning and delivery of a planned dose distribution compared to titanium.^6^ While radiation physicists are developing metal suppression protocols to limit metallic artifact in radiation planning, future studies are needed to compare the clinical outcomes of titanium hardware with these emerging protocols compared to CFRP.

The advantage in radiation dose calculations with CFRP implants Is most relevant with primary spinal tumors. There is significant variability between surgeons and institutions in the use of post-operative radiation for primary tumors. However, when radiation is pursued, it typically involves proton beam radiation or carbon ion therapies which are particularly sensitive to high Z material such as titanium where dose calculations can be significantly affected.^6,15,16,29^ This is particularly relevant due to the potential curative intent for these procedures, longer life expectancy compared to spinal metastases, and the need for long-term radiographic follow-up.^8^

The benefits of CFRP in the setting of particle therapy have been reported by Mastella et al.^14^ Utilizing dosimetry studies they demonstrated that CFRP had less beam perturbation and dose degradation compared to titanium. Additionally, this work demonstrated significant particle therapy dose reductions 4 cm behind titanium screws compared to 10% with CFRP (14). Work from Muller et al arrived at similar results utilizing retrospective planning with CT data from 5 patients with titanium and CFRP implants. While volumetric arc therapy (VMAT) dose distributions were equivalent, the presence of CFRP with intensity modulated proton therapy (IMPT) plans resulted in a smaller dosimeteric impact on HU uncertainties compared to titanium.^5^ The value of CFRP in the setting of proton therapy was similarly investigated by Poe et al, who demonstrated a 90% artifact reduction with proton beam radiation compared to titanium.^30^

Despite the increasing utilization of CFRP for spinal malignancies, there are several limitations and drawbacks that have been documented. While authors have cited multiple perceived advantages of CFRP and along with their safety profile, there have been no clinical studies demonstrating an objective oncologic benefit of CFRP in patients with spinal tumors. To date, reported case series continue to be limited in size, have relatively short follow-up, lack prospective comparison with titanium implants, and utilize different CFRP systems which each have their structural differences and technical nuances. For example, Icotec pedicle screws are coated with titanium along the threads to facilitate osseous integration at the pedicle interface and increase pullout resistance.^31^ Additionally, the polyaxial screw head is made of titanium and the screw tip has a tantalum marker to facilitate fluoroscopic visualization.^6,31^ In contrast, the screw head with Carboclear pedicle screws are CFRP and radiolucent. Further studies will be needed to robustly evaluate the clinical consequences of these differences on radiation planning, treatment, radiographic monitoring, and construct stability.

Additional general challenges with CFRP implants include lack of haptic feedback with screw insertion, the requirement to tap prior to insertion in blastic bone, and reduced visualization both during screw placement and on post-operative imaging. This makes confirmation of adequate screw positioning within the vertebral body and subsequent monitoring for implant failure challenging. Limitations also exists due to lack of posterior cervical carbon fiber screws and carbon fiber rod options. Carbon fiber rods are packaged with a pre-bent curvature that cannot be bent intraoperatively which may limit their utilization in all cases. CFRP implants have also been cited to have lower tensile strength which may contribute to failure with compressive and shear forces.^32^ Last, CFRP implants are associated with a significantly higher cost when compared to titanium screws, prompting concern regarding their financial justification.^33^

## Future Directions

Based on the extant literature, carbon fiber posterior instrumentation is safe and has a clinical efficacy that is at least equivalent to titanium.^13^ Additionally, while there are numerous reported advantages to CFRP, there are several recurring themes that highlight important areas of future investigation. Addressing these points would help identify the optimal utilization of CFRP into normal spinal oncology workflows, facilitate improved patient selection, and highlight the impact of CFRP on improved oncologic outcomes.

The majority of cases in our systematic review reported carbon fiber screws in the setting of spinal metastases. However, the surgical intent, post-operative radiation workflow, and duration of follow-up varies significantly between metastatic disease and primary tumors of the spine. Future studies will need to examine primary and metastatic spinal tumors separately and systematically compare the influence of titanium and CFRP hardware on proton and photon radiation planning, radiation administration, detection of local disease recurrence, quantifying overall survival, and monitoring for fusion, long-term hardware failure, and spinal deformity. Attention should be directed at developing standardized surgical protocols that involve pre-operative planning, intraoperative technique, and post-operative radiation planning and follow-up. For instance, identifying high risk cases amongst primary and metastatic tumors where CFRP could be particularly valuable for detection of recurrent/foraminal disease. Additionally, it is possible that cases can be discussed and imaging reviewed with radiation oncology prior to surgery in order to determine if CFRP implants would be valuable in the post-operative radiation planning setting. Additionally, bone quality varies significantly between primary spinal tumors and metastases. In primary tumors, adjacent levels are typically not affected by tumor. In contrast, the bone quality of metastatic spinal tumors is frequently poor and can vary significantly between histologies. Moreover, osteopenia is a significant concern amongst the elderly oncologic population. As a result, attention should be directed at the decision-making process to perform cement augmentation for CFRP screws to enhance screw anchorage.

An additional recurring theme is the need for long-term prospective studies to determine clinical benefit. To date, the only study formally comparing CFRP and titanium implants was reported by Cofano et al. While the authors did not observe a significant difference in complications between either implant type, the study was limited to 78 patients (38 with CFRP fixation and 42 with titanium fixation). Due to the lack of studies that include a sufficient number of patients with adequate follow-up to compare CFRP and titanium implants, this should be a priority in the future. This type of prospective study, ideally with a matched titanium cohort, should incorporate emerging metal suppression protocols with radiation planning and emphasize long-term follow-up to clarify whether there is a clinical benefit of CFRP on local control secondary to improved radiation administration and enhanced detection of local recurrence. A study of this nature would also provide sufficient time to monitor for fusion, overall spinal alignment, and resolution of oncologic and mechanical back pain. Finally, our systematic review included studies from two separate CFRP systems that vary in their incorporation of titanium components, resulting in a heterogenous cohort of reviewed patients. While considering prospective studies, it would be particularly helpful to account for CFRP implants from different vendors and determine their relative clinical equipoise.

A significant concern with CFRP implants is their significant cost differential compared to titanium. For instance, surveyed contract pricing from multiple US hospitals reflected a 4-5-fold increase in cost of fenestrated carbon fiber pedicle screws compared to titanium. Future studies will need to include cost-benefit-analyses of CFRP compared to titanium. As part of this process, it will be critical to account not only for implant costs but also for downstream expenses such as potential impact on operative time, complication rates, and the cost of radiation planning between CFRP and titanium cases. Additionally, a comparison between the accuracy of spinal cord contouring on MRI and CT myelogram in the setting of CRFP implants could lead to a change in practice, avoiding the need for an invasive CT myelogram, expediting the delivery of treatment, and reducing costs. It is also possible to consider the use of hybrid CFRP/titanium constructs in order to reduce costs. In this scenario, carbon fiber screws could be placed at the adjacent level above and below the site of separation surgery while titanium is used at the remaining levels. While this approach has the potential of reducing costs and maintaining radiolucency at the surgical site, the biomechanical implications of this hybrid approach are unclear and may limit the ability to detect epidural tumor creep outside the surgical level.

A final area of future work relates to technology development. While Boriani et al has reported alternative strategies for CFRP cervical instrumentation,^17^ there are no CFRP lateral mass screws or occipital plates currently available. Future work to increase the breadth of sizes and screw types will be a significant advance in the use of CFRP implants along the entire craniospinal axis. Additionally, while CFRP rods are available and were widely used in the studies of the systematic review, they come in various pre-bent shapes that cannot be modified. However, there are clinical scenarios where a pre-manufactured CFRP rod does not exist for the needed spinal construct. Future efforts should be devoted to this limitation, developing additional rod contours and providing a widely accessible a pre-operative workflow similar to 3-D printed implants that would facilitate expedited pre-manufactured CFRP rods unique to needs of each surgical case.

In an effort to investigate the interest of CFRP implants as an alternative to titanium for spinal reconstruction, the North American Spine Society section of Spinal Oncology surveyed its members regarding the clinical utility, practice patterns, and recommended future directions of radiolucent spinal implants.^34^ Respondents had mixed opinions regarding these implants and their ability to provide clinically meaningful benefits for the detection of disease recurrence and improvement of post-operative radiation. Additionally, several concerns were cited including high cost, low availability, limited posterior cervical options, suboptimal design, inability to contour rods, and decreased ease of use compared to titanium. Taken together, 73.3% of respondents were hesitant to adopt a radiolucent strategy. Many institutional experiences and reviews have now been published on CFRP implants which suggest CFRP is a safe implant with several perceived advantages that appear most significant in the context of primary tumors receiving proton or carbon ion therapy. However, these survey results along with our systematic review of the literature highlight the need of the international spinal oncology community to move towards larger, robust clinical studies to identify patients who will receive the greatest benefit from CFRP implants; investigate the oncologic, economic, and biomechanical implications of CFRP implants on patient care; and demonstrate the overall impact on clinical outcomes.
